# Excitotoxicity Triggered by Neurobasal Culture Medium

**DOI:** 10.1371/journal.pone.0025633

**Published:** 2011-09-28

**Authors:** Joshua Hogins, Devon C. Crawford, Charles F. Zorumski, Steven Mennerick

**Affiliations:** 1 Departments of Psychiatry, Washington University School of Medicine, St. Louis, Missouri, United States of America; 2 Department of Anatomy & Neurobiology, Washington University School of Medicine, St. Louis, Missouri, United States of America; 3 Graduate Program in Neuroscience, Washington University School of Medicine, St. Louis, Missouri, United States of America; University of Houston, United States of America

## Abstract

Neurobasal defined culture medium has been optimized for survival of rat embryonic hippocampal neurons and is now widely used for many types of primary neuronal cell culture. Therefore, we were surprised that routine medium exchange with serum- and supplement-free Neurobasal killed as many as 50% of postnatal hippocampal neurons after a 4 h exposure at *day in vitro* 12–15. Minimal Essential Medium (MEM), in contrast, produced no significant toxicity. Detectable Neurobasal-induced neuronal death occurred with as little as 5 min exposure, measured 24 h later. D-2-Amino-5-phosphonovalerate (D-APV) completely prevented Neurobasal toxicity, implicating direct or indirect *N*-methyl-*D*-aspartate (NMDA) receptor-mediated neuronal excitotoxicity. Whole-cell recordings revealed that Neurobasal but not MEM directly activated D-APV-sensitive currents similar in amplitude to those gated by 1 µM glutamate. We hypothesized that L-cysteine likely mediates the excitotoxic effects of Neurobasal incubation. Although the original published formulation of Neurobasal contained only 10 µM L-cysteine, commercial recipes contain 260 µM, a concentration in the range reported to activate NMDA receptors. Consistent with our hypothesis, 260 µM L-cysteine in bicarbonate-buffered saline gated NMDA receptor currents and produced toxicity equivalent to Neurobasal. Although NMDA receptor-mediated depolarization and Ca^2+^ influx may support survival of young neurons, NMDA receptor agonist effects on development and survival should be considered when employing Neurobasal culture medium.

## Introduction

Primary dissociated neuronal cultures remain an important mainstay of neuroscience research. Various media have been optimized for neuronal survival and development in cell culture. Among these, Neurobasal enjoys widespread popularity. Its use has extended well beyond its original optimization for survival of embryonic hippocampal neurons over a few days in cell culture [1]. Despite these extended uses, the effects of Neurobasal on most aspects of cell development remain incompletely characterized.

In dissociated hippocampal cultures tested at day *in vitro* 12–15, we found that routine media switches (feedings) with fresh, supplement-free Neurobasal resulted in significant cell death. Similar cell death was not observed with another standard medium, Minimal Essential Medium (MEM). We hypothesized that a single component of Neurobasal was responsible for the neuronal loss. A combination of whole-cell recording and toxicity studies revealed that L-cysteine, a known bicarbonate-dependent NMDA receptor agonist [2], was responsible for the cell loss. The commercial formulation of Neurobasal contains 26 times the amount of L-cysteine cited in the original report of optimized Neurobasal medium [1]. We conclude that NMDA agonist activity of Neurobasal medium should be taken into account in its use in neuronal cell culture.

## Results

### Neurobasal is toxic to mature hippocampal neurons

**Figure 1:**
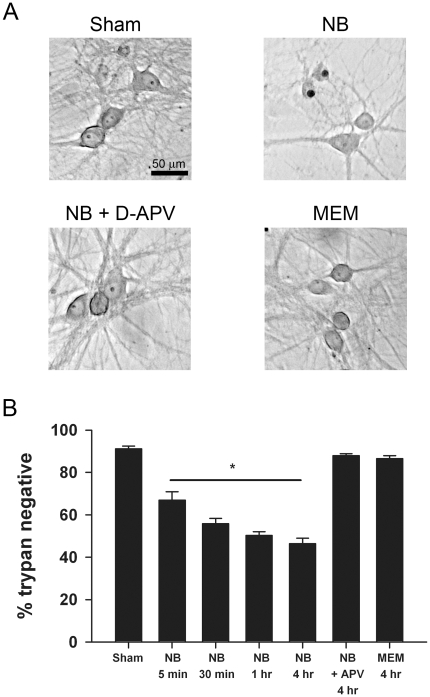
Fresh Neurobasal is neurotoxic to cultured hippocampal neurons. **A**. Photomicrographs from a single experiment showing toxicity resulting from fresh Neurobasal (NB) incubation. Upper left: brightfield image from a control sham medium exchange stained with trypan blue 24 h after the challenge. Upper right: a field from a dish incubated in fresh Neurobasal for 4 h. Pyknotic, trypan positive nuclei with remnants of cell bodies are present. Lower left: 50 µM D-APV, a competitive NMDA receptor antagonist, was included in the fresh Neurobasal medium and protected neurons from Neurobasal toxicity. Lower right: A field from a dish incubated for 4 h in MEM, matched to Neurobasal for inorganic salts, osmolarity, pH, glucose, and glycine concentrations. **B**. Summary of experiments like that of panel A showing the effects of Neurobasal (NB) exposure over various incubation times. MEM incubation had minimal effect (N = 4 independent platings, asterisk indicates a main effect of Neurobasal compared with sham condition; p<0.01, one-way ANOVA).

Neurobasal culture medium is widely used to support survival of many different neuronal populations. Therefore, we were surprised to find that incubation in fresh Neurobasal medium, in the absence of L-glutamine or other supplements, killed mature (cultured for 12–14 days) hippocampal neurons. To characterize this phenomenon we incubated neurons in Neurobasal medium for various time periods (Figure 1A, B). After the allotted time, the cells were returned to their original conditioned medium and incubated normally for 24 h. Cell counts of trypan blue-positive neurons the following day revealed consistent Neurobasal-induced cell loss (Figure 1A). With a sham medium switch we found only mild attritional cell death consistent with previous observations in our cultures (91±1.3% healthy neurons) [3]. Neurobasal incubation for 4 h killed approximately half of our cultured neurons (Figure 1B).

Hippocampal neurons are known to be quite sensitive to NMDA receptor-mediated excitotoxicity [4]–[7]. Furthermore, microscopic appearance of cells following Neurobasal incubation (acute swelling) seemed to suggest excitotoxicity. Consistent with the idea that over-stimulation of glutamate receptors mediates Neurobasal-induced neuronal death, 50 µM D-APV, a competitive NMDA receptor antagonist, fully protected cells from Neurobasal-induced toxicity (88±1.0% cell survival, Figure 1B, p>0.05 compared with sham control). Another culture medium, MEM, failed to produce significant cell death after a 4 h incubation (Figure 1B; 87±1.4% survival, p>0.05 compared with sham control), suggesting that a specific ingredient unique to Neurobasal is responsible.

### Neurobasal elicits a direct, NMDA receptor-dependent current

Neurobasal could contain an ingredient that directly activates glutamate receptors, or some component that depolarizes neurons through a different mechanism, leading to toxic secondary release of glutamate. To distinguish these possibilities, we recorded Neurobasal-elicited currents from voltage-clamped, synaptically isolated hippocampal neurons grown in microcultures. To maintain appropriate pH of solutions in the absence of incubator CO_2_, we diluted Neurobasal (or control MEM solution) by 50% in standard HEPES buffered recording bath solution. The cells were voltage-clamped at −30 mV using the whole-cell patch-clamp technique. The relatively depolarized clamp potential was chosen to limit Mg^2+^ blockade and thereby increase detection of NMDA receptor-mediated currents [8]. We chose MEM as a control medium based on its lack of neurotoxicity (Figure 1).

**Figure 2:**
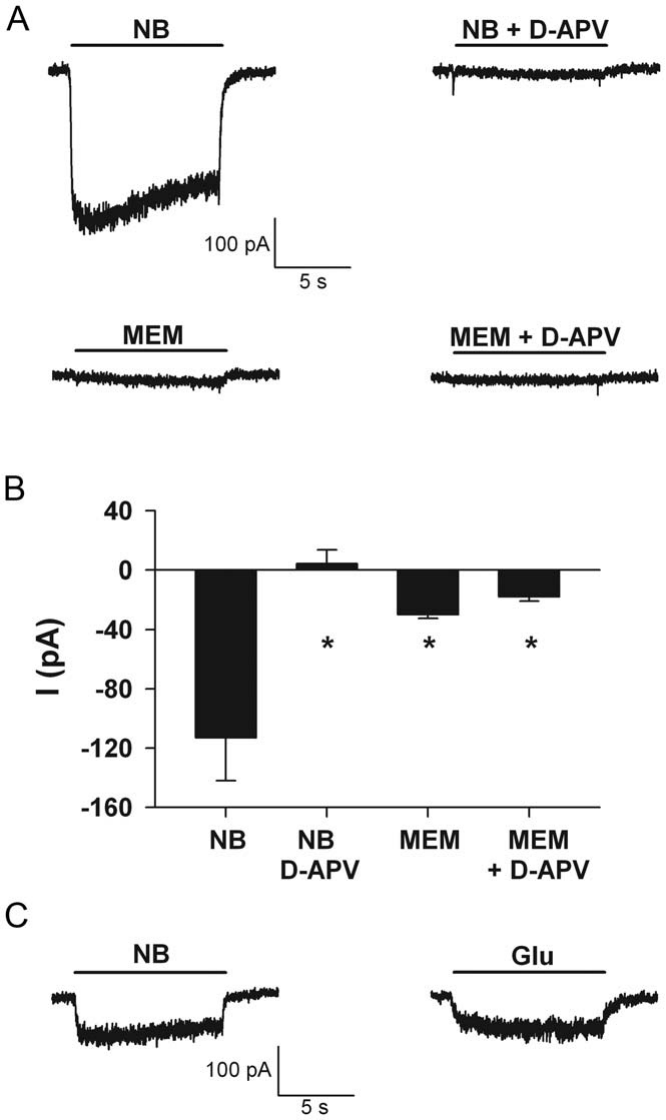
Acute Neurobasal application (diluted 50% in recording saline) generates a D-APV-sensitive current in synaptically isolated hippocampal neurons grown in microculture. **A**. Representative currents from a cell demonstrating the response to 50% Neurobasal (NB) in the absence and presence of 50 µM D-APV. MEM application (50% dilution) produced minimal current. In this and subsequent figures, cells were voltage-clamped at −30 mV to relieve Mg^2+^ block of the NMDA receptor channel. Scale bar applies to all traces. **B**. Summary of the steady-state current amplitudes from 5 cells. Asterisk designates p<0.05 compared with sham (unpaired, two-tailed t-tests with Bonferroni correction for multiple comparisons). **C**. Representative traces from a cell demonstrating similar current amplitude generated by acutely applied Neurobasal (NB) diluted to 50% in standard saline and 0.5 µM glutamate (Glu) added to BBS. The scale bar applies to both traces. The faster onset kinetics and offset kinetics of Neurobasal-gated currents compared with glutamate-gated currents were a consistent finding (see Results). These calibrations suggest that undiluted Neurobasal contains a low-affinity agonist, equivalent to 1 µM glutamate.

Neurobasal elicited inward currents in all cells tested, and these currents were sensitive to D-APV (Figure 2A, B). MEM did not gate equivalent currents (Figure 2A, B). Taken with the results in Figure 1, this suggests that a component present in Neurobasal, but not in MEM, directly activates NMDA receptors in hippocampal neurons. For comparison, we determined the glutamate concentration required to gate an equivalent NMDA receptor-mediated response. In the presence of NBQX to block AMPA/kainate receptors, we found that 0.5 µM glutamate activated a steady-state current nearly equivalent to 50%-diluted Neurobasal medium (Figure 2C). For this experiment we used bicarbonate buffered saline (BBS) diluted by 50% in regular saline recording solution for glutamate exposure. BBS contained equivalent salts and bicarbonate buffer to Neurobasal. The steady-state currents gated by the 50% dilution of Neurobasal were 96±13% of glutamate-gated currents in the same cells (N = 4). Therefore, we judge that undiluted Neurobasal contains the equivalent of 1 µM glutamate. However, the kinetics of Neurobasal-activated currents were always faster than the kinetics of currents activated by glutamate (offset time constant 78±27 ms for Neurobasal currents versus 460±110 ms for glutamate-gated currents, p<0.05, Student's paired, 2 tailed *t* test). Because current offset time constant is related to the dissociation rate constant of agonist from receptor [9], this result suggests that the agonist present in Neurobasal medium has a lower affinity for the NMDA receptor than the natural neurotransmitter glutamate.

### L-cysteine, a component of Neurobasal, gates an NMDA receptor-mediated current

**Figure 3:**
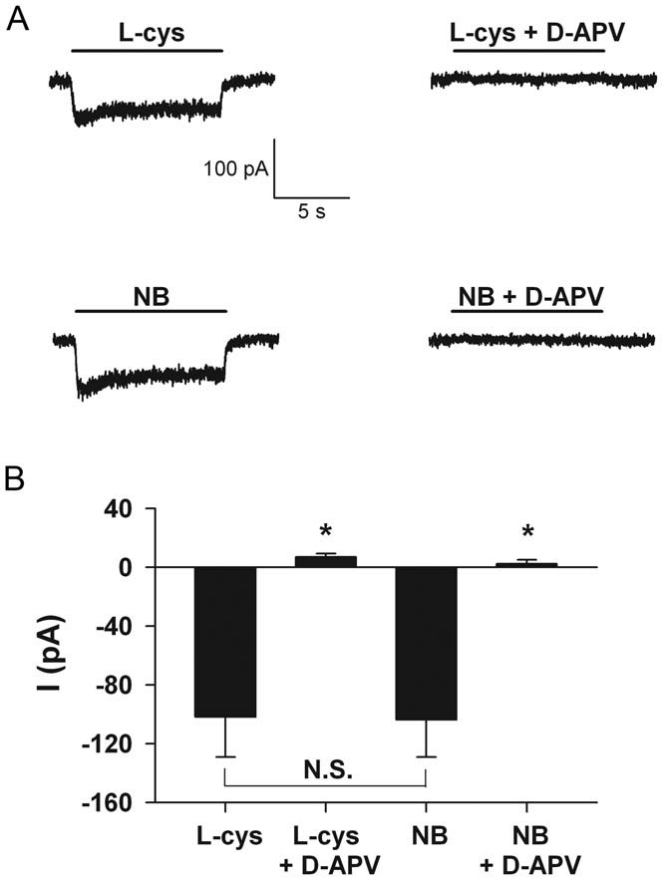
L-cysteine, a component of Neurobasal, elicits a D-APV-sensitive current similar to Neurobasal. **A**. Representative currents from a hippocampal neuron showing the response to acute application of 130 µM L-cysteine (L-cys) and 50% Neurobasal (NB). The current was blocked by co-application of 50 µM D-APV. Acute application of L-cysteine in BBS and Neurobasal generated similar D-APV-sensitive current amplitude and kinetics. The calibration bar applies to all traces. **B**. Summary of steady-state current amplitude from 7 cells. Asterisk designates p<0.05 compared with current amplitude in the absence of D-APV (unpaired, two-tailed t-tests with Bonferroni correction for multiple comparisons). N.S. designates no statistical significance (p>0.05 using paired, two-tailed *t* tests).

L-cysteine is a bicarbonate-sensitive NMDA receptor agonist with excitotoxic potential [2]. The bicarbonate sensitivity of L-cysteine may result in part from carbamate formation [10]. Neurobasal, a bicarbonate-buffered medium, contains 260 µM L-cysteine, a concentration that approaches levels shown to be excitotoxic in *ex vivo* chick retina [2]. Notably, L-cysteine is absent from MEM, which failed to generate an NMDA receptor-mediated current (Figure 2A, B) or toxicity (Figure 1A, B). To determine whether L-cysteine generates currents similar to those gated by Neurobasal, we recorded responses to Neurobasal and L-cysteine in the same neurons. We applied 130 µM L-cysteine in BBS and compared responses to those of 50% Neurobasal. L-cysteine generated D-APV-sensitive currents very similar in kinetics (3±19% faster time constant of deactivation, N = 4) and amplitude to those of Neurobasal (Figure 3A, B). These results are strong evidence that the L-cysteine in Neurobasal is responsible for NMDA agonist activity and likely for excitotoxicity.

### L-cysteine causes excitoxicity similar to Neurobasal

**Figure 4:**
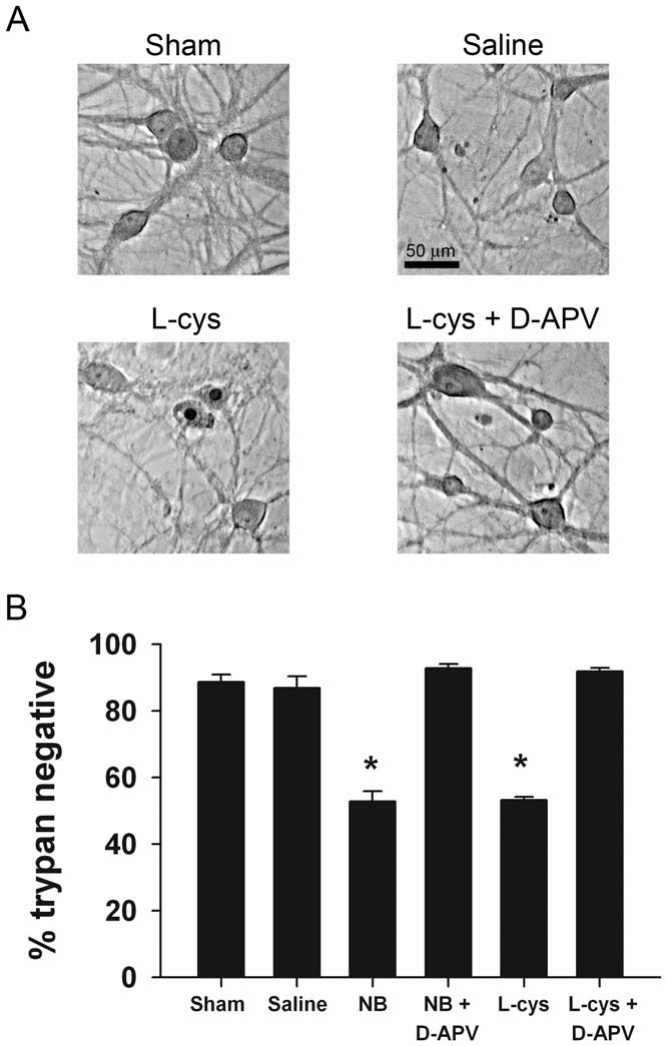
L-cysteine and Neurobasal generate similar toxicity. **A**. Photomicrographs from a single experiment showing toxicity as a result of L-cysteine (260 µM) incubation. Upper left: brightfield image from a sham medium exchange control. Upper right: A field from a dish incubated for 4 h in BBS (saline). Lower left: a field from a dish exposed to BBS plus 260 µM L-cysteine for 4 h. Trypan-positive nuclei with remnants of cell bodies are present. Lower right: 50 µM D-APV protected against L-cysteine-induced toxicity. **B**. Summary of experiments like that of panel A compared with Neurobasal (NB) incubation in sibling cultures. Neurotoxicity of 260 µM L-cysteine in BBS is similar to Neurobasal toxicity, and both are blocked by co-incubation with 50 µM D-APV (N = 4 independent experiments on independent platings). Asterisk designates p<0.05 compared with sham (unpaired, two-tailed t-tests with Bonferroni correction for multiple comparisons).

It is possible that L-cysteine interacts with another ingredient of Neurobasal (in addition to bicarbonate) over the time course of toxicity experiments to trigger cell loss. To test the idea that L-cysteine alone accounts for Neurobasal toxicity, we compared toxicity of Neurobasal to that of L-cysteine applied in BBS (Figure 4A). As in the experiments represented in Figure 1, after the 4 h exposure we returned the cells to their original medium and allowed them to incubate normally for an additional 24 h. BBS with 260 µM L-cysteine caused cell death equivalent to exposure to fresh Neurobasal over the same time period (Figure 4B). Also, the cell death from both manipulations was prevented by the addition of 50 µM D-APV (Figure 4B).

L-cysteine or L-cysteine derivatives may affect metabotropic glutamate receptors (mGluRs), which might interact with NMDA receptor function to increase excitotoxic damage [11]–[13]. To test this we co applied 260 µM L-cysteine with 25 µM LY341495, a concentration at which this drug is a broad-spectrum mGluR inhibitor [14], [15]. We found no difference in neuronal damage between exposure to L-cysteine alone and L-cysteine plus LY341495 (sham media exchange: 92±1.5% healthy, 260 µM L-cysteine alone: 54±5.1% healthy, and L-cysteine plus antagonist: 52±3.0% healthy; comparison of L-cysteine conditions: p = 0.82, N = 3). Thus, inhibition of mGluRs during L-cysteine exposure did not protect from excitotoxic damage. This suggests, with our previous experiments, that toxicity is mostly initiated through L-cysteine's action at NMDA receptors.

Neurobasal is typically used with supplements such as B-27 (Invitrogen) for optimized neuronal development. B-27 may contain protective factors, such as antagonist polyamines, which might weaken L-cysteine's effects [1]. However, we found that co-application of B-27 supplement (2% vol/vol) with L-cysteine to BSS for 4 h failed to alter L-cysteine toxicity (L-cysteine alone: 54±5.1% healthy; L-cysteine+B-27: 53±1.2% healthy, p>0.05, 2-tailed Students t-test, N = 3). Taken together, our results strongly suggest that L-cysteine is responsible for Neurobasal neurotoxicity and toxicity is not antagonized by supplement factors.

## Discussion

Our results identify L-cysteine as a specific component of Neurobasal that mediates NMDA receptor-dependent neurotoxicity in mature hippocampal cultures. Although the original formulation of Neurobasal was designed to support optimal growth of developing neuronal cultures, its use has become much wider. When employing Neurobasal medium, investigators should consider the NMDA receptor agonist effects, including possible excitotoxicity. These results also add to a growing literature implicating a number of amino acids that can mediate excitotoxicity, an important participant in a variety of neurological and neuropsychiatric disorders [2], [6], [16]–[21].

Excitatory neurotransmission is required for normal brain function, and NMDA receptor stimulation with calcium influx is required for neonatal and immature brain development [22], [23]. Culture media such as Neurobasal are optimized for this, and we employ this media successfully in our culture system of dissociated hippocampal neurons. In our hands, partial media exchanges in younger cultures (see Methods) are well tolerated. Vulnerability to excitotoxic injury progresses as cultures age and parallels the expression of glutamate/NMDA receptors and the formation of synaptic connections [24]–[26]. In neurons with a high density of glutamate receptors, achieved with synaptic maturity, excitatory neurotransmission is a double-edged sword. The link between the excitatory amino acid neurotransmitter glutamate and neuronal excitotoxic brain damage is well established [4], [6]. In addition to L-glutamate, other amino acids are active at glutamate receptors, particularly NMDA type receptors, and have the potential to cause excitotoxicity under certain conditions [2], [21], [27].

Routine media exchanges or feedings with fresh Neurobasal in mature dissociated hippocampal cultures caused excitotoxicity, and we sought to determine the component responsible. We showed that standard MEM, similar to Neurobasal but with fewer ingredients, did not cause cell death (Figure 1A, B). When scrutinizing the differences in components of Neurobasal and MEM, we discovered that several amino acids, including L-cysteine, are notably absent from MEM. We also discovered that the original formulation of Neurobasal contained 26 times less L-cysteine than the present commercial formulation [1]. Systemically administered L-cysteine causes widespread neuronal excitotoxic damage in rodents, and *in vitro* experiments revealed that high micromolar concentrations represent a threshold for excitotoxic damage to retinal neurons [2]. The concentration of L-cysteine (260 µM) in the current commercial formulation of Neurobasal is in the lower range capable of producing excitotoxicity in chick retina [2]. L-cysteine therefore became a prime candidate in mediating the cell death we observed in hippocampal cultures in response to fresh Neurobasal. We established that the ability of Neurobasal to gate NMDA receptors was equivalent to that of approximately 1 µM glutamate (Figure 2C). These effects are at the low end of glutamate's ability to activate NMDA receptors (approximately 20% of maximum) [27]. Furthermore 260 µM L-cysteine is on the low end of the concentration range that produces excitotoxicity in other systems [2]. Nevertheless, this level of receptor stimulation clearly generated excitotoxicity of hippocampal neurons.

The mechanisms underlying excitotoxic neuronal cell death are complex. Although the mechanism of L-cysteine excitotoxicity is not fully understood, L-cysteine neurotoxicity depends on bicarbonate and occurs within physiologic pH [2], [28]. It has been proposed that α-carbamate formation may be key to toxicity of L-cysteine and other bicarbonate-sensitive excitotoxins [10]. L-cysteine could contribute to excitotoxicity in neuronal networks in a number of other ways. Our experiments ruled out an important contribution of metabotropic glutamate receptors and showed that direct activation of NMDA receptor likely initiates toxicity. However, our results do not exclude a number of secondary effects to NMDA receptor stimulation [12]. Through NMDA receptor-dependent depolarization, L-cysteine could enhance synaptic glutamate release, thereby contributing to additional receptor overstimulation [29]. In addition, L-cysteine derivatives such as the oxidized form cystine are substrates for the astrocytic glutamate/cystine transporter, which would elevate extracellular glutamate levels [30], [31]. L-cysteine may inhibit uptake of extracellular glutamate as well [12], [28], [32].

Excitotoxicity from oxygen and glucose deprivation or exogenous NMDA receptor stimulation has both acute and delayed components. Acute effects include cation and anion flux and cell swelling. Ca^2+^-dependent delayed effects through intracellular cascades ultimately also contribute to cell death [17], [33]. We measured cell death 24 hours after the insult in order to capture both the acute and delayed effects. Factors such as L-cysteine uptake, L-cysteine metabolism, NMDA receptor desensitization, or other protective/adaptive mechanisms may explain the apparent plateau of cell death with 4 h exposure time (Figure 1) [6], [7], [16].

L-cysteine-gated NMDA receptor activation may have other unappreciated effects on cellular and synaptic development. For instance, mild depolarization of young neurons by NMDA receptor activation may actually promote survival in some circumstances [34]. NMDA receptor activation also has important effects on the efficacy of synaptic transmission, perhaps via receptor desensitization [35], [36], and the ability of synapses to generate plasticity [37]. Tonic depolarization also can exert short-term and longer-lived homeostatic effects on excitability, synaptic function, and network properties [38]–[40]. These and other effects of sustained NMDA receptor activation should be considered when Neurobasal medium or other L-cysteine-containing media are employed for culture work.

In summary, we have shown that L-cysteine, at the concentration present in commercially formulated Neurobasal medium, mediates an NMDA receptor-dependent excitotoxic reaction in dissociated hippocampal neuronal cultures. This NMDA agonist effect, as well as other effects of L-cysteine-mediated NMDA receptor activation on developing neurons, should be taken into account when employing Neurobasal and other media with high L-cysteine concentrations. Further study is needed to determine more clearly the mechanism by which L-cysteine elicits bicarbonate-dependent NMDA receptor activation and excitotoxicity. This mechanism could have important consequences in the understanding of neuropathological processes involving excitotoxins.

## Materials and Methods

### Ethics Statement

All studies were carried out in accordance with the recommendations in the Guide for the Care and Use of Laboratory Animals of the National Institutes of Health. The protocol was approved by the Washington University Animal Care and Use Committee, protocol approval number 20080234.

### Cell culture

Hippocampal cultures were prepared as described previously [41]. In brief, under isoflurane anesthesia, postnatal day 0–3 male and female rat pups were decapitated and hippocampi were incubated with papain, mechanically dissociated, and plated at 650 cells/mm^2^ on collagen substrate for mass cultures. Plating medium consisted of MEM with Earle's salts (Invitrogen, catalog number 11090) supplemented with heat-inactivated horse serum (5%), fetal bovine serum (5%), 17 mM glucose, 400 µM glutamine, 50 U/ml penicillin, and 50 g/ml streptomycin. Cultures were maintained at 37°C in a humidified incubator with 5% CO_2_/95% air. Cytosine arabinoside at 6.7 µM was added at 3–4 d after plating to inhibit glial proliferation. A half medium exchange was also performed with Neurobasal medium (Invitrogen) plus B27 supplement with L-glutamine, 50 U/ml penicillin, and 50 g/ml streptomycin 4–5 days after plating. Young cultures tolerate this half media exchange well possibly because of a low density of NMDARs (see Discussion). Cultures were maintained at 37°C in a humidified incubator with 5% CO_2_/95% air and cells were used for experiments at 12–15 days *in vitro*.

Electrophysiological experiments were performed on microisland cultures prepared on microstamped dishes. To prepare these cultures, dishes were precoated with 0.15% agarose type II-A (Sigma), and allowed to dry. After drying, collagen was applied to a polydimethylsiloxane stamp [42], which had been cleaned using a PDC-001 plasma cleaner (Harrick Plasma). The collagen was then stamped onto the dish and exposed to ultraviolet light for 45 min. Cortical astrocytes from 4 day-old rat pups were established on the stamped dishes in plating medium (above). After astrocytes were established for 3–6 d, hippocampal neurons obtained as above were seeded at 25 cells/mm^2^ onto the astrocyte microislands.

### Toxicity Experiments and Media Exchange

Experiments were conducted on mass cultures at 12–15 days *in vitro*. The experimental media used were fresh filtered Neurobasal (Invitrogen) medium without L-glutamine, MEM, or a bicarbonate/HEPES buffered saline solution (BBS) containing (in mM) 111.81 mM NaCl, 26.19 mM NaHCO_3_, 5.33 mM KCl, 1.8 CaCl_2_, 0.4 mM glycine, 0.814 MgCl_2_, 25 glucose, and 10 mM HEPES buffer. This BBS recipe was adopted to replicate salt concentrations contained in Neurobasal and was used where noted for media exchange and toxicity experiments. The bicarbonate concentration has been shown previously to be important as a co-factor for L-cysteine effects at NMDA receptors [2], [28]. All media used were matched for pH (7.25), osmolarity (310 mosmol), and temperature (37°C). When sham medium exchange was performed as a control, the conditioned medium was removed for several seconds and then returned to the culture dish to simulate a medium exchange. For experimental medium exchange, the cells' original conditioned medium was removed and replaced with fresh Neurobasal, MEM, or BBS where noted. All incubation media were free of serum or supplements. After the exposure to the fresh, experimental media, the cells were returned to their original medium and allowed to recover in a humidified 37°C incubator until a cell death assay was performed 24 h later. In toxicity experiments where indicated, 260 µM L-cysteine, the concentration in Neurobasal supplied by the manufacturer, was added to the BBS. When tested for neuroprotection against neurotoxicity, 50 µM D-APV was added to the media.

### Cell Death Assay

The cell death assay was conducted as previously described [7]. To assess cell death, trypan blue dye (Sigma) was used. 24 h after the insult, culture media were removed and replaced with 1 mL of 0.4% trypan blue dissolved in phosphate buffered saline. Cells were incubated in dye at 37°C for 5 min and washed with phosphate buffered saline at room temperature. Cultures were then fixed with 4% paraformaldehyde and 0.2% glutaraldehyde at room temperature. Cells were visualized with a 20× objective using both phase-contrast and brightfield microscopy to confirm healthy neuronal profiles (phase-contrast) and verify trypan blue uptake (brightfield). The experimenter was always naïve to the experimental condition. The total numbers of dead and intact neurons were counted and expressed as a percentage of total cells. The average of ten microscope fields for each condition was treated as a single data point for statistical purposes. Representative brightfield images were obtained with a 40× objective using a CoolSnap ES2 camera (Photometrics) and Metamorph software (MDS).

### Electrophysiology

Solitary microisland neurons were used for all electrophysiology experiments. Using synaptically isolated neurons helped us to distinguish the direct activation of NMDA receptors from secondary receptor activation resulting from network glutamate release. Control and experimental conditions were always performed on sibling cultures from the same litter and plating and on the same day of recording.

Whole-cell recordings were performed using an Axopatch 200B amplifier (Molecular Devices) and a Digidata 1322 acquisition board (Molecular Devices). The whole-cell pipette solution contained the following (in mM) 130 cesium methane sulfonate, 4 NaCl, 0.5 CaCl_2_, 5 EGTA, 10 HEPES, and 16% glucose (pH 7.25). Electrodes had resistances of 3–5 M Ω, and access resistance was less than 15 MΩ.

For experiments depicted in Figure 2 A and B, the culture medium was exchanged for a standard recording bath solution containing the following (in mM): 138 NaCl, 4 KCl, 2 CaCl_2_, 0 MgCl_2_, 0.01 glycine, 10 glucose, 10 HEPES, and 0.01 glycine, pH 7.25. For all other electrophysiological experiments (Figures 2C, 3, and 4), the bath solution was BBS diluted 50% in standard recording saline. Solutions were applied to recorded cells with a local, multi-barrel pipette system. Junction current exchange times at an open tip were <150 ms. When applying culture medium to neurons during whole-cell recording, media were diluted by 50% with standard recording saline solution.

### Data Analysis

Data analysis was performed in Excel (Microsoft) and Sigma Plot 10.0 (Systat Software). Student's unpaired *t* test, Student's paired *t* test, or one-way ANOVA was used to test for significance where noted. Where indicated, the Bonferroni correction for multiple comparisons was used. For toxicity experiments, values for *n* in the text and figure legends represent independent experiments on separate cultures, but *n* represents the number of neurons in electrophysiology experiments. Data are expressed as mean ± standard error of the mean in the text and figures.
